# Inhibitory Effect of Gualou Guizhi Decoction on Microglial Inflammation and Neuron Injury by Promoting Anti-Inflammation via Targeting mmu-miR-155

**DOI:** 10.1155/2021/2549076

**Published:** 2021-08-17

**Authors:** Haixia Hu, Xinghua Zhong, Xinjun Lin, Jinbo Yang, Xiaoqin Zhu

**Affiliations:** ^1^Academy of Integrative Medicine, Fujian University of Traditional Chinese Medicine, Fuzhou, China; ^2^Fujian Key Laboratory of Integrative Medicine on Geriatrics, Fuzhou, China; ^3^Institute of Integrated Chinese and Western Medicine, Fujian University of Traditional Chinese Medicine, Fuzhou, China

## Abstract

Gualou Guizhi decoction (GLGZD) treatment exerts neuroprotective effects and promotes spasticity following ischemic stroke. However, the molecular mechanism of GLGZD treatment on ischemic stroke remains unclear. Our previous study indicated that GLGZD ameliorates neuronal damage caused by secondary inflammatory injury induced by microglia. In the present study, we investigate the potential mechanism of GLGZD treatment on neuron damage induced by neuroinflammation via mmu-miR-155 in vitro. The HT22 cell line and the BV2 cell line were exposed to oxygen/glucose-deprive (OGD) conditions; the conditioned medium was prepared using the supernatants from OGD-stimulated BV2 cells after pretreating with GLGZD. Cell viability was determined by MTT assays; levels of released inflammatory cytokines were assessed using the BioPlex system. mmu-miR-155 and its targeting genes were detected using real-time reverse transcription polymerase chain reaction (RT-PCR). The expression of anti-inflammatory proteins was evaluated by Western blotting. DAPI staining was used to test the apoptotic cells. Our results showed that GLGZD pretreatment significantly induced IL10 release and decreased the production of TNF-*α*, IL6, and IFN-*γ*. In addition, GLGZD markedly attenuated mmu-miR-155 expression and its downstream SOCS1, SMAD2, SHIP1, and TAB2 expression levels. The DAPI-stained apoptotic cell death and caspase-3 activation in HT22 cells exposed to the conditioned medium were reversed by GLGZD treatment. Our findings suggested that GLGZD pretreatment downregulates the mmu-miR-155 signaling, which inhibits microglial inflammation, thereby resulting in the suppression of neuron apoptosis after OGD stress. The underlying mechanisms may provide the support for GLGZD treatment of cerebral ischemic injury.

## 1. Introduction

Ischemic stroke remains a leading cause that challenge public health worldwide. Microglia are the major immune cells in the nervous system, which can be activated and involved in the neuropathological development [1, 2]. The molecules released from injured brain tissue provoke microglial activation and generate a ring of cytokines, some of which exacerbate primary brain damage, and others exert anti-inflammatory effects [3–5]. mmu-miR-155, a highly expressed miRNA in the CNS, is reported to be a neuroinflammatory regulator targeting different mediators, SHIP1, SOCS1, SMAD2, and TAB2, which involve in inflammatory balance [6, 7]. Mmu-miR-155 promotes inflammatory reaction by suppressing SHIP1, SOCS1, SMAD2, and TAB2 expressions, all of which inhibit inflammation by decreased expressions of proinflammatory cytokines and increased expressions of anti-inflammatory cytokines [8, 9]. Given the significance of mmu-miR-155 in the regulation of neuroinflammation, the treatment targeting mmu-miR-155 in ischemic cerebral injury will be identified as a promising therapy for ischemic stroke.

Gualou Guizhi decoction (GLGZD) is a commonly used traditional Chinese medicine and prescribed in the “Essentials from the Golden Cabinet,” which is a traditional Chinese medical work written by Zhongjing Zhang in Han Dynasty. It has been used for clinical treatment of spasticity following ischemic stroke and mainly used at The Fujian University of the TCM Affiliated Second People's Hospital (Fuzhou, China). GLGZD's recipe includes Radix Trichosanthis, Ramulus Cinnamomi, Radix Paeoniae Alba, Radix Glycyrrhizae, Rhizoma Zingiberis Recens, and Fructus Ziziphi Jujubae. The neuroprotective effect of GLGZD may involve a number of different mechanisms, including reduced inflammatory cytokine levels, an antioxidation effect, and the modulation of Glu levels. Based on our previous study, GLGZD has been shown to possess multiple pharmacological properties to reduce inflammation and protect neuron from severe injury. However, the underlying mechanisms of treatment remain unclear. The present study attempted to explore the anti-inflammatory effects of GLGZD on OGD-induced BV2 activation regulated by mmu-miR-155 signaling and the neuroprotective role of GLGZD in HT22 cell apoptosis stimulated by microglial conditioned medium; this study will further reveal the underlying possible mechanisms of GLGZD treatment for cerebral ischemia injury.

## 2. Materials and Methods

### 2.1. GLGZD Preparation

The prescribed herbal medicine were obtained from Guoyitang Drug Store (Fuzhou, China). The decoction ingredients and GLGZD extracts preparation were applied in this experiment according to our previous study [10, 11]. The working concentrations for treatment of GLGZD were 100–400 *μ*g/ml.

### 2.2. HT22 and BV2 Cell Culture

Immortalized mouse hippocampal neuron HT22 cells and the murine BV2 microglial cell line were grown in high-glucose Dulbecco's modified Eagle's medium (DMEM, Gibco, Carlsbad, USA) supplemented with 10% fetal bovine serum (FBS, Gibco) and 1% penicillin/streptomycin. The cells were maintained at 37°C in a humidified atmosphere of 5% CO_2_. BV2 cells were pretreated by GLGZD (100 *μ*mol/L) for 2 h and then stimulated with different treatments.

### 2.3. Oxygen-Glucose Deprivation Model

BV2 cells were cultured in media deprived from oxygen and glucose to induce OGD condition and then placed in an incubator (H45, Whitely, England) with humidified atmosphere containing 1% oxygen, 94% nitrogen, and 5% CO_2_ at 37°C. All cells were subjected to 4 h OGD followed by incubation of the 24-hour reperfusion period in the normal culture medium. Controls were incubated with the normal culture condition and the identical time as the OGD culture.

### 2.4. GLGZD Treatment and Preparation of Microglial Conditioned Medium (MCM)

GLGZD was extracted and diluted with the culture medium to various concentrations: 100, 200, and 400 *μ*g/ml. The BV2 cells were pretreated with different concentration of GLGZD for 2 h and subsequently exposed to OGD injury. The MCM was derived from the supernatant of BV2 cells subjected to OGD stress and pretreated with or without GLGZD. The MCM was collected and added to HT22 cultures.

### 2.5. 3-[4, 5-Dimethyl-thiazol-2-yl]-2,5-diphenyltetrazolium Bromide (MTT) Assay

HT22 cells were seeded at the density of 3 × 10^4^ cells/well in a 96-well plate and then cultured overnight, and then, the medium was replaced with the MCM for additional 24 h incubation. BV2 cells were plated onto 96-well plates (5 × 10^4^ cells/well) and pretreated with the indicated concentrations of GLGZD for 2 h before OGD stress. The cell viability of BV2 cell subjected to OGD and HT22 exposed to MCM were measured by MTT assay. Briefly, 100 *μ*L of 0.05% MTT reagent (Sigma, St. Louis, MO) was added to each well and incubated at 37°C for 4 h followed by 100 *μ*L of DMSO solution to dissolve the formazan crystals. The absorbance at 570 nm was evaluated using a microplate reader (ElX-880, BioTek Inc., Winooski, VT). The data were expressed as percentages of the control.

### 2.6. Detection of Cytokines by the BioPlex System

After OGD injury and GLGZD treatment, the BV2 cell culture supernatant was harvested to measure the cytokine concentrations. BioPlex inflammation-related ELISA kits (#M50-0KCAF0Y, BioRad Laboratories, Hercules, CA, USA) were used to measure the secretion levels of a ring of cytokines according to the manufacturer's protocol, including TNF*α*, IL6, IFN-*γ*, and IL10. Briefly, add 50 *μ*l diluted beads to each well of the assay plate, and then, wash the plate with BioPlex wash buffer. After that, add 50 *μ*l standards, samples, and controls to incubate on the shaker at 850 rpm for 30 min at RT. The detection antibodies were added to each well followed by washing the plate three times. The plate was incubated at 850 rpm for 30 min at RT. Then, the streptavidin-phycoerythrin was added to each well and incubated at 850 rpm for 10 min at RT. After resuspending the beads in assay buffer and shaking at 850 rpm for 30 sec, the results were determined using BioPlex 200 system (BioRad Laboratories, Hercules, CA, USA) at 450 nm.

### 2.7. Real-Time PCR

Total RNA was isolated using TRIzol R reagent (Invitrogen, Carlsbad, CA, United States). Reverse transcription to synthesize cDNA was performed using the RevertAid First Strand cDNA Synthesis Kit (Fermentis Life Science, Ontario, Canada). cDNA template was used for the quantitative real-time PCR amplification. Specific primers were designed, and the primer sequences for each gene are as follows: mmu-miR-155-5p: Fwd 5′-CAGTTTGTGGAACGGTGCTG-3′; Rev 5′-TGGTAGGGTCATCGGGTTCT-3'; SOCS1: Fwd 5′-CTGCGGCTTCTATTGGG GAC-3′, Rev 5′-AAAAGGCAGTCGAAGGTCTCG-3′; SMAD2: Fwd 5′-CATCAGCCAATGGCAAGTGAA-3′, Rev 5′-AGAACAGGGTCTGCATCCATCATA-3′; SHIP1: Fwd 5′-CAGGGATGAAGTACAACTTGCC-3′; Rev 5′-TCTCCTTCCTGACTCTTGACA-3′; TAB2: Fwd 5′-CGATCAGCTGTTGCGAGCGCTGCAC-3′; Rev 5′-GTGCAGCGCTCGCAACAGCTGATCG-3′; and GAPDH: Fwd 5′-CACATTGGGGGTAGGAACAC-3′; Rev 5′-ACCCAGAAGACTGTGGATGG-3′. RT-PCR was conducted using the following condition: 94°C for 30 s, 55°C for 45 s, and 72°C for 1 min for a total of 35 cycles, 72°C for 10 min. GAPDH was served as an internal control for normalization. The relative expression level of the PCR products was analyzed using the 2^(−∆∆Ct)^ method.

### 2.8. Western Blot Analysis

After exposure to different treatments, cells were collected and lysed in RIPA-containing protease and phosphatase inhibitors (Roche, Penzberg, Germany) followed by centrifugation at 12,000 g for 5 min at 4°C. The supernatant was collected, and the protein concentration was assessed using the BCA assay (Beyotime, Biotechnology, Beijing, China). 50 *μ*g of proteins was separated by SDS-PAGE electrophoresis and transferred to polyvinylidene difluoride (PVDF) membranes (BioRad, Munich, Germany). The membranes were then blocked in 5% nonfat milk for 1 h at room temperature to prevent nonspecific binding and incubated overnight at 4°C with primary antibodies against SOCS1, SMAD2, SHIP1, and TAB2 (1 : 1000, Cat. No. 68631 for SOCS1, Cat. No. 5339 for SMAD2, Cat. No. 2725 for SHIP1, and Cat. No. 3745 for TAB2, Cell Signaling Technology, Beverly, MA). Afterward, the membranes were washed with TBS/T (TBS with 0.05% Tween 20) and incubated with the secondary antibody IgG (1 : 5000, Cat. No. 91196, HRP Conjugate, Cell Signaling Technology, Beverly, MA) at room temperature for 1 h. The immune-reactive proteins were visualized using the enhanced chemiluminescence (ECL) reagent (P0018, Beyotime Biotechnology). *β*-Actin (anti-*β*-actin antibody, cat. no. sc-47778, Santa Cruz Biotechnology, Inc. Dallas, TX, USA), was used as an internal reference. The data were normalized against the internals reference and then expressed as fold changes compared to the controls, quantified by using BioRad ChemiDoc™ XRS Image Lab Software.

### 2.9. DAPI Staining for Nuclei

HT22 cells were cultured on 6-well culture plates at a density of 3 × 10^5^ cells/mL and treated with MCM as described above. After fixation with 4% paraformaldehyde at room temperature for 20 min, the cells were stained with 10 mg/mL 6-diamidino-2-phenylindole dihydrochloride (DAPI; Beyotime Biotechnology, Shanghai, China) for 15 min. The formation of fragmented or condensed DNA was determined as typical characteristic of apoptotic cells. Representative photographs were taken by a fluorescence microscope (Leica Microsystems, Wetzlar, Germany) at 100× and 200× magnification. The cell fluorescence was measured using image J software. The percentage of apoptotic cells in total cells was calculated.

### 2.10. Detection of Caspase-3 Activity

The caspase-3 activity test was carried out according to the instructions of caspase-3 activity assay kit (lot no. C1116, Beyotime Biotechnology, Shanghai, China). In brief, the cells were centrifugated and collected for protein extraction and quantification. After that, the Ac-DEVD-pNA (acetyl-Asp-Glu- Val-Asp p-nitroanilide) was added to extract protein to incubate for 90 min till production of yellow PNA (p-nitroaniline). Finally, the absorbance at 405 nm was detected by a microplate reader (BioTek 8008, Bad Friedrichshall, Germany), and the caspase-3 activity was determined according to the standard curve.

### 2.11. Statistical Analysis

Three independent experiments were conducted; all results were expressed as mean ± SD. Statistical analysis was determined by one-way analysis of variance (ANOVA) using SPSS 20.0 software. Differences were considered statistically significant at *p* < 0.05.

## 3. Results

### 3.1. Protective Effects of GLGZD Pretreatment on OGD-Induced Injury in BV2 Cells

**Figure 1:**
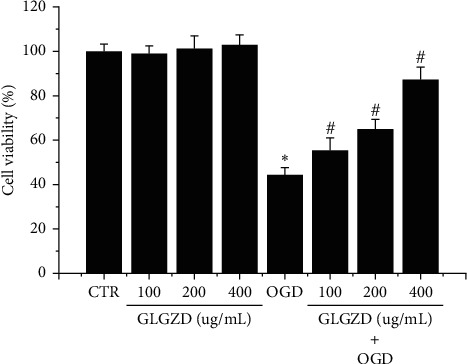
effect of GLGZD on the viability of BV2 cells subjected to OGD stimulation. The MTT assay was used to determine the viability of BV2 cells. All data were expressed as mean ± SD of three independent experiments performed in triplicate. ^*∗*^*P* < 0.05 vs. control (CTR) and ^#^*P* < 0.05 vs. OGD condition.

As shown in Figure 1, the viability of BV2 cells exposed to OGD insult was measured using the MTT assay, which indicated that exposure to OGD for 4 h followed by additional 24 h of reperfusion that resulted in significant decrease of cell viability. However, when compared with the OGD group, pretreatment with GLGZD could significantly inhibit the OGD induced injury in BV2 cells in a concentration dependent manner (*p* < 0.05). Furthermore, the BV2 survival was not obviously affected by GLGZD alone at the concentration from 100 to 400 *μ*g/mL.

### 3.2. GLGZD Pretreatment Regulated OGD-Induced Cytokines Production in BV2 Cells

**Figure 2:**
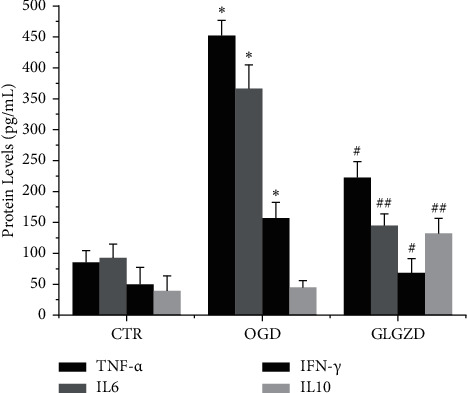
The impact of GLGZD on the expression levels of pro and anti-inflammatory cytokines in OGD-stimulated BV2 cells. The protein levels of pro and anti-inflammatory cytokines were analyzed by BioPlex multiple ELISA. Data are expressed as mean ± SD from three independent experiments. ^*∗*^*P* < 0.05 vs. control and ^#^*P* < 0.05, ^##^*P* < 0.01 vs. OGD condition.

To investigate the anti-inflammatory function of GLGZD, we performed multiELISA to assess the release of inflammatory cytokines. In our study (Figure 2), compared to the control group, the production of TNF-*α*, IL6, and IFN-*γ* in the culture medium supernatants was significantly enhanced by OGD stress (*p* < 0.05) while reduced by GLGZD pretreatment (*p* < 0.05 and 0.01). In contrast, low levels of IL10 productions in the OGD group were increased significantly in the GLGZD (200 *μ*g/mL) group (*p* < 0.01).

### 3.3. Effect of GLGZD on Expression Levels of mmu-miR-155 and Its Targeting Genes in BV2 Cells under OGD Condition

**Figure 3:**
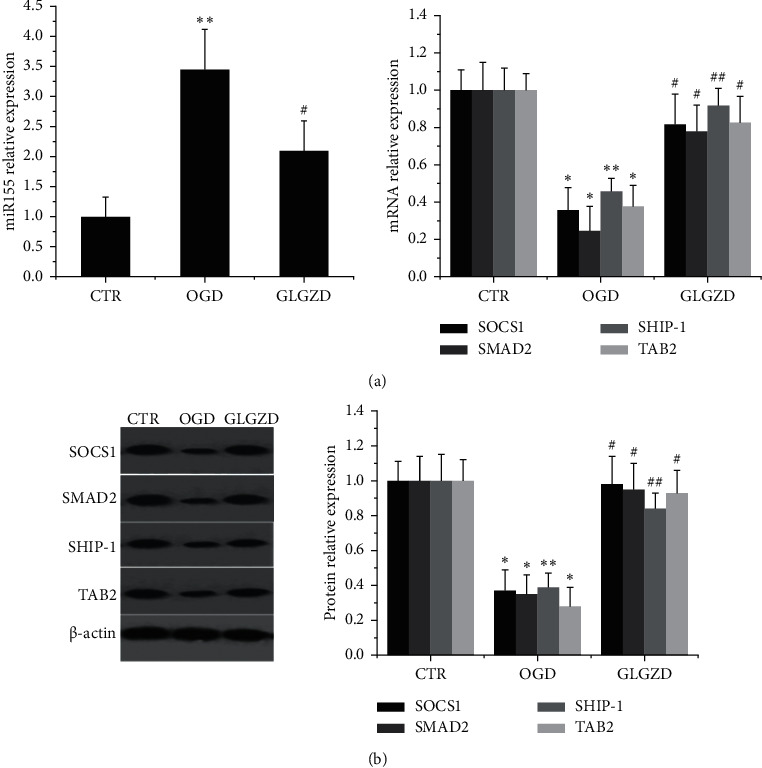
GLGZD prominently restricts the level of mmu-miR-155 and promotes the expression of its targeting meditators. (a) The mRNA levels of mmu-miR-155 and SOCS1, SMAD2, SHIP1, and TAB2 measured by RT-PCR and quantitative assessment performed by normalizing to the level of internal control GAPDH. (b) The protein expression level of SOCS1, SMAD2, SHIP1, and TAB2 assessed by Western blotting assay and normalized to the level of internal control *β*-actin. *β*-Actin was used for normalization, and the intensity of bands was quantified by densitometric analysis. All values represent mean ± SD of three independent experiments. ^*∗*^*P* < 0.05, ^*∗∗*^*P* < 0.01 vs. control and ^#^*P* < 0.05, ^##^*P* < 0.01 vs. OGD condition.

mmu-miR-155 is a key microRNA that involved cell inflammation poststroke, and previous results indicated that its levels are obviously raised in activated microglia. The present study showed that mmu-miR-155 and its downstream genes were obviously influenced by GLGZD pretreatment in BV2 subjected by OGD. As shown in Figure 3(a), compared with the control group, there was an obvious increase in mmu-miR-155 (*p* < 0.01) and decrease in SOCS1, SMAD2, SHIP1, and TAB2 in the OGD group (*p* < 0.05 and *p* < 0.01). However, GLGZD treatment significantly suppressed the upregulation of mmu-miR-155 (*P* < 0.05) and increased SOCS1, SMAD1, SHIP1, and TAB2 mRNA expression induced by OGD (*p* < 0.05 and *p* < 0.01). Simultaneously, the protein alteration in different groups were consistent with the mRNA expression results (*p* < 0.05 and *p* < 0.01, Figure 3(b)).

### 3.4. GLGZD Protected HT22 Cells from the Neurotoxic Microglial Conditioned Medium Induced by OGD

**Figure 4:**
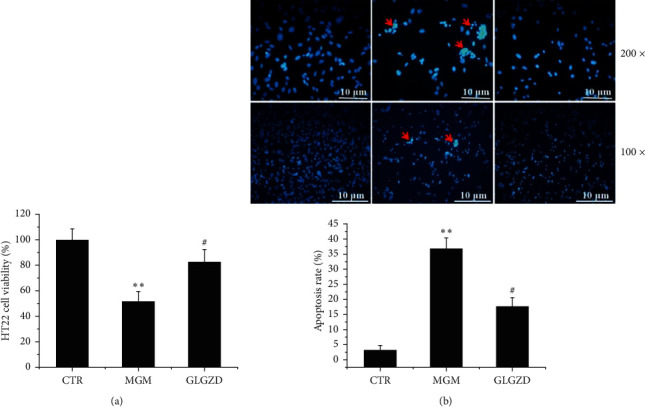
The neuroprotection of GLGZD on the MCM-induced cytotoxicity in HT22 cells. (a) The cell viability of HT22 cells tested by MTT assay. (b) The apoptotic HT22 cells presented DAPI staining. Arrowheads indicted apoptosis cells and the apoptosis rate was calculated. All data are expressed as mean ± SD of three independent experiments. ^*∗∗*^*P* < 0.01 vs. control and ^#^*P* < 0.05 vs. OGD condition.

Microglial conditioned medium (MCM) was established and added to HT22 for incubation. Afterward, to examine the neuroprotective potential of GLGZD, the HT22 cell viability was tested using MTT assay, and the cell apoptosis was assessed by DAPI staining. As shown in Figure 4(a), the conditioned medium from activated microglia induced decrease in HT22 cell viability compared with the control group; however, treatment with GLGZD at 200 *μ*g/mL concentration restored the cell survival partially (*p* < 0.05, Figure 4(a)). Accordingly, DAPI staining was utilized for detecting HT22 cells of nuclear morphometry; the presence of chromatin condensation and nuclear shrinkage is indicative of the occurrence of apoptosis. Our results showed that there were more apoptotic cells in the MCM-treated group, and GLGZD pretreatment reduced the number of apoptotic cells significantly (*p* < 0.05, Figure 4(b)).

### 3.5. GLGZD Ameliorated Caspase-3 Activity in MCM-Induced HT22 Cells

**Figure 5:**
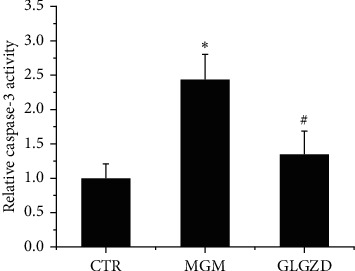
GLGZD reverses the activity of caspase-3 in MCM-induced HT22 cells. The caspase-3 activity was assayed by a microplate reader at absorbance of 405 nm. All results were presented as mean ± SD of three independent experiments. Results represent 3 independent experiments. ^*∗*^*P* < 0.05 vs. control and ^#^*P* < 0.05 vs. OGD condition.

Caspase-3, which belongs to caspase family, plays a key role in the process of apoptosis, including chromatin condensation and DNA fragmentation [12]. A significant increase in caspase-3 activity was observed following MCM stimulation in HT22 cells compared with that of the control group (*p* < 0.05). In contrast, pretreatment with GLGZD reversed the influence of OGD injury and significantly reduced caspase-3 activity in HT22 cells exposed to MCM (Figure 5, *p* < 0.05).

## 4. Discussion

Ischemic stroke is a neurological disorder resulted from loss of blood supply leading to neuronal damage. Dead neurons generate danger-associated molecular patterns (DAMPs) triggering excessive inflammatory responses [13, 14]. Microglia are the resident immune cells in the brain and play a central role in the postischemic inflammation. Activated microglia release a wide range of inflammatory mediators including proinflammatory cytokines exacerbating brain injury. On the other hand, anti-inflammatory cytokines are produced by microglia conversely promoting brain repair [15, 16]. Develop novel immunomodulatory approaches to improve mortality and functional outcome of those inflicted with ischemic stroke. Thus, developments in identifying immunomodulatory approaches will contribute to limit neuronal injury and improve poststroke functional impairment.

Ample evidence indicate that miRNAs potentially involve in many biological processes at the posttranscriptional level, including cell apoptosis and inflammation [17, 18]. Among various miRNAs, miR-155 is considered as the key regulator in postischemic brain inflammation [19]. In addition, miR-155 can exert both pro and anti-inflammatory roles by targeting different mediators, such as SHIP1, SOCS1, SMAD2, and TAB2. When ischemia occurs, the expression of mmu-miR-155 was significantly upregulated, and inhibiting mmu-miR-155 could reduce the secretion of TNF-*α*, IL6, IL1*β*, and IFN-*γ* induced by neuroinflammation [20, 21].

Fewer side effects and multitarget therapy are the advantages of traditional Chinese medicine (TCM) in the clinical treatment of stroke. GLGZD is a widely used and classical TCM for approximately more than 10 years as an effective treatment for spasticity poststroke. The molecule mechanism may correlate with the neuron protection from serious damage through antioxidation and anti-inflammation [22, 23]. Moreover, GLGZD promotes neurological functional recovery by increasing neuron proliferation and enhancing axonal regeneration following focal cerebral ischemia. In our previous study, GLGZD exhibited significant anti-inflammatory effects on LPS-stimulated BV2 cells. Here, we further explore the underlying mechanism of neuroprotective potential of GLGZD pretreatment in the cultured BV2 cells and HT22 cells exposed to OGD injury.

In our present study, we established an OGD model to investigate the function of GLGZD in BV2 and HT22 cells. The results showed that the addition of GLGZD significantly reversed the decreased BV2 cell viability induced by OGD. Furthermore, GLGZD pretreatment blocked the upregulated expression levels of TNF-*α*, IL6, and IFN-*γ* and promoted the expression levels of IL10 induced by OGD insult in BV2 cells. To test whether GLGZD treatment is involved in mediating mmu-miR-155 signaling in inflammatory reaction, mRNA and protein levels of the specific targeting mediators of mmu-miR-155 were detected by RT-PCR and Western blotting assays. We observed a dramatic upregulation in mmu-miR-155 expression in vitro model by exposing BV2 cells to OGD conditions. Simultaneously, the expression levels of its target mediators were affected obviously by GLGZD. Among these mediators, SOCS1, SMAD2, SHIP, and TAB2 enhance the production of anti-inflammatory cytokines and reduce the generation of proinflammatory cytokines; however, GLGZD treatment can promote their effects on inflammation significantly. These observations demonstrated that GLGZD not only play a protective role in microglia but also inhibit microglial overactivation. Moreover, the impact of GLGZD was dependent on mmu-miR-155-induced signal in microglia. Then, to further examine the effect of GLGZD on the MCM-treated HT22 cells, the results suggested that the neuron apoptotic cells and caspase-3 activity evoked by OGD insult were decreased by GLGZD treatment. Taken together, our findings clearly revealed that the neuroprotective properties of GLGZD were involved in negative regulation of inflammation via mmu-miR-155 and antiapoptotic role after the induction of OGD.

To sum up, our data confirmed the protective effect of GLGZD on neuron injury induced by inflammatory and apoptotic effects mediated by mmu-miR-155 signaling, thereby providing experimental evidence of GLGZD to alleviate brain damage and improve functional recovery for ischemic stroke therapy.

## Data Availability

The datasets analyzed to support the findings of this study are available from the corresponding author upon request.

## Abbreviations

GLGZD: Gualou Guizhi decoction
IL1*β*: Interleukin-1*β*
IL6: Interleukin-6
IL10: Interleukin-10
OGD: Oxygen-glucose deprivation
TCM: Traditional Chinese medicine
IFN-*γ*: Interferon-gamma
TNF-*α*: Tumor necrosis factor-*α*
DAPI: 4′, 6-Diamidino-2-phenylindole
SOCS1: Suppressors of cytokine signaling 1
SMAD2: Signal transduction molecule 2
SHIP1: Src homology 2 (SH2) domain containing inositol polyphosphate 5-phosphatase 1
TAB2: TGF-beta activated kinase 1 (TAK1)/MAP3K7 binding protein 2.
